# Lessons learned: Characteristics of first-year COVID-19 hospital outbreaks

**DOI:** 10.1017/ash.2022.184

**Published:** 2022-05-16

**Authors:** Sophie Solar, Emily Blake, Sithembile Chithenga, Mefruz Haque, Anitra Denson, Renee Zell, Jennifer Steppe, Anil Mangla, Preetha Iyengar

## Abstract

**Background:** At the start of the COVID-19 pandemic, the DC Department of Health (DC Health) mandated new case reporting for early outbreak detection: (1) weekly healthcare personnel (HCP) absenteeism line lists indicating staff absent for confirmed or suspected SARS-CoV-2, (2) daily line lists of all SARS-CoV-2–positive inpatients, and (3) hospital contact tracing. Between March 27, 2020, and December 31, 2020, DC Health detected 36 confirmed and 14 suspected hospital outbreaks, of which only 18% (8 confirmed and 1 suspect) were known to the affected hospital. DC Health learned which outbreaks warranted early or aggressive intervention by tracking outbreak characteristics across its jurisdiction. This allowed prioritization of during surges when it was difficult for DC Health and hospital staff to investigate every outbreak. **Methods:** Potential outbreaks in short-stay and inpatient rehabilitation hospitals were flagged after identifying SARS-CoV-2 hospital-onset (HO) inpatients or staff clusters on line lists. Variables of interest in line lists included specimen collection and hospital admission dates, units or departments, and patient contact. Facility contact tracing by infection preventionists further verified epidemiological links among cases. Outbreak details were systematically tracked in a locally developed REDCap database and were analyzed if they had an initial case, outbreak start date, or an investigation start date in 2020. Frequency procedures, SQL statements, and date calculations were computed using SAS Enterprise Guide version 8.3 software. **Results:** Confirmed outbreaks had an average of 6.92 (range, 0–32) HCP and 2.58 (range, 0–22) patient cases, with 69% being confirmed-HO cases and 31% probable HO. Moreover, 53% of confirmed outbreaks occurred in the following departments: cardiac, behavioral health, intensive care, and environmental services (EVS)/facilities. All of these departments had recurrent outbreaks. Behavioral health, medical and cardiac units had the highest number of patient cases. On average, confirmed outbreak investigations lasted 24.6 days, with outbreaks prolonged in the ICU (40.25 days) and the medical unit (37.67 days). Top triggers for investigations ultimately classified as confirmed outbreaks were (1) positive symptomatic HCP, (2) confirmed-HO cases, and (3) exposures from positive HCP. **Conclusions:** The dynamic nature of COVID-19 created challenges in detecting and responding to hospital outbreaks. Developing a low-resource outbreak tracking system helped identify outbreak types and triggers that warranted early or aggressive interventions. Understanding the characteristics of hospital outbreaks was critical for maximizing infection control resources during surges of infectious disease outbreaks, such as COVID-19. Hospitals or local health departments could adapt this system to meet their needs.

**Funding:** None

**Disclosures:** None

